# The sweet side of vimentin

**DOI:** 10.7554/eLife.35336

**Published:** 2018-03-07

**Authors:** Natasha T Snider, Nam-On Ku, M Bishr Omary

**Affiliations:** 1 Department of Cell Biology and PhysiologyUniversity of North Carolina at Chapel HillChapel HillUnited States; 2 Interdisciplinary Program of Integrated OMICS Biomedical science, Graduate SchoolYonsei UniversitySeoulRepublic of Korea; 3 Department of Molecular & Integrative PhysiologyUniversity of Michigan Medical SchoolAnn ArborUnited States; 4 Department of MedicineUniversity of Michigan Medical SchoolAnn ArborUnited States

**Keywords:** O-GlcNAc, intermediate filaments, cell migration, Chlamydia trachomatis, glycosylation, chemical biology, Human

## Abstract

A protein modification called O-linked glycosylation regulates the interactions between vimentin molecules under normal conditions, and the ability of *Chlamydia* bacteria to replicate after they infect cells.

**Related research article** Tarbet H, Dolat L, Smith T, Condon B, O’Brien TE, Valdivia R, Boyce M. 2018. Site-specific glycosylation regulates the form and function of the intermediate filament cytoskeleton. *eLife* **7**:e31807. doi: 10.7554/eLife.31807

The cytoskeleton is a critical cellular structure that is formed by three distinct protein networks: microtubules, which are involved in intracellular transport; actin filaments, which are involved in cell motility; and intermediate filaments, which provide structural and mechanical support (Pegoraro et al., 2017). Although intermediate filaments are the least studied of the three, more than 80 tissue-specific human diseases are known to be associated with mutations in intermediate filament genes (http://www.interfil.org).

The networks formed by intermediate filaments are highly dynamic and individual filaments also undergo constant remodeling (Robert et al., 2016). Moreover, various post-translational modifications of the filaments, such as phosphorylation and glycosylation, play key roles in regulating their dynamics (Snider and Omary, 2014). A particularly important modification for intermediate filaments is O-linked glycosylation (Hart et al., 2007), which involves the addition of a sugar-like molecule called GlcNAc (short for N-acetylglucosamine) to specific serine (Ser) or threonine (Thr) amino acid residues in multiple intermediate filament proteins, including vimentin (Slawson et al., 2008). However, there is much about O-linked glycosylation that is not well understood (Bond and Hanover, 2015).

Now, in eLife, Michael Boyce of Duke University School of Medicine and co-workers – including Heather Tarbet as first author – report on the role of O-linked glycosylation in the formation of vimentin intermediate filaments (Tarbet et al., 2018). They also shed light on the process by which *Chlamydia trachomatis* bacteria 'hijack' vimentin molecules to form a cage and stabilize the cytoplasmic vacuoles in which they replicate.

A vimentin molecule, like all other intermediate filament proteins, has three domains (Figure 1), and it is known that the head domain contains several amino acids that are sites for both glycosylation and phosphorylation (Izawa and Inagaki, 2006; Slawson et al., 2008; Snider and Omary, 2014). To form an intermediate filament, vimentin molecules first form dimers which then assemble to form tetramers that then associate laterally and longitudinally to assemble into mature 10nm-wide filaments of varying lengths. Tarbet et al. studied how three post-translational modification sites might be involved in the interactions between vimentin molecules. Two of these sites, Ser34 and Ser39 (numbering is inclusive of methionine at position 1), are known to be sites for both phosphorylation and glycosylation, whereas the third – Ser49 – is thought to be a site for glycosylation, but not for phosphorylation.

**Figure 1.**
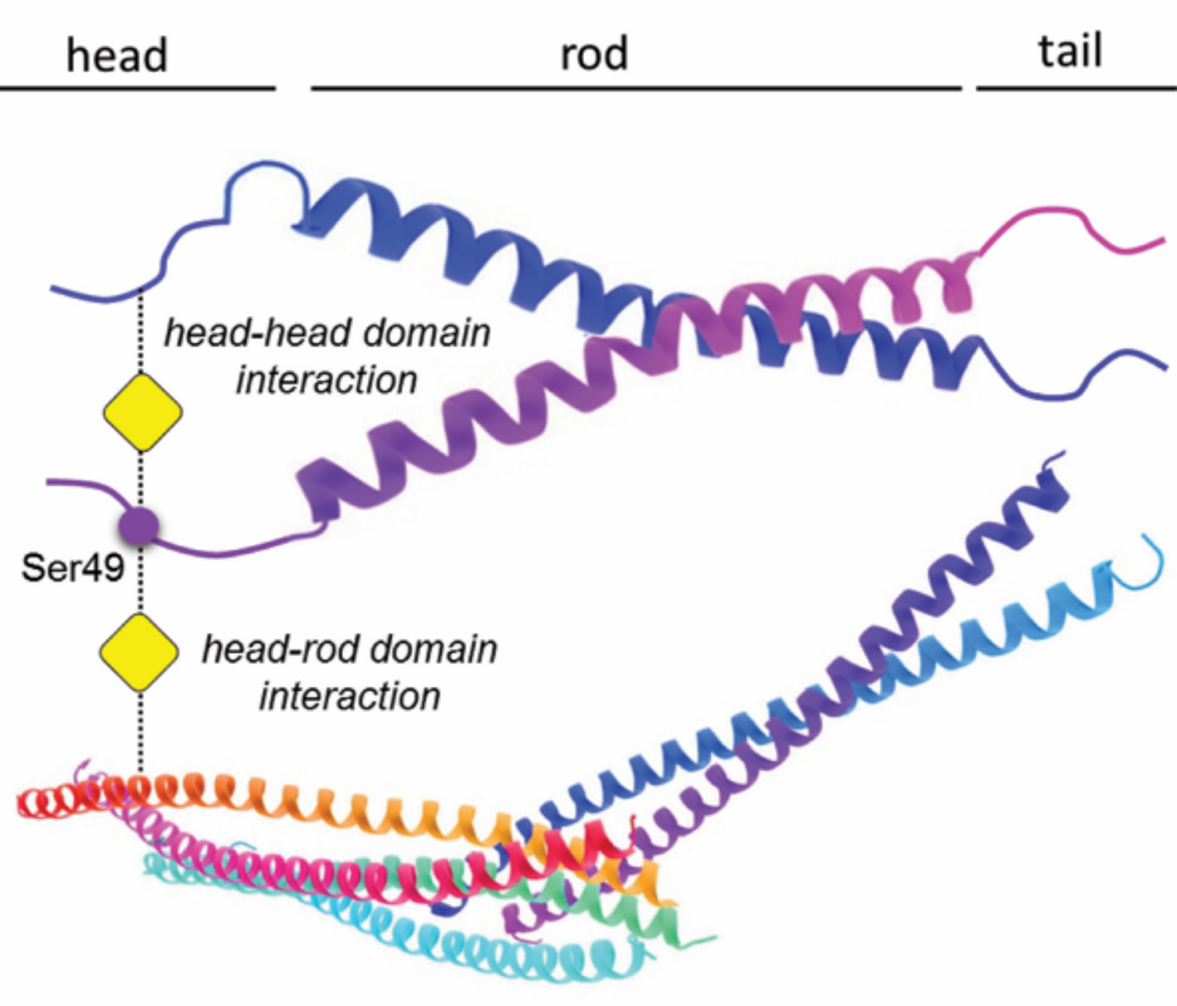
Glycosylation of a serine residue in the ‘head’ domain of vimentin promotes interactions between vimentin molecules. A vimentin molecule has three domains: the head, the rod (which is α-helical), and the tail. Tarbet et al. show that the glycosylation of the Ser49 residue in the head domain (indicated by the addition of the yellow square) promotes the formation of vimentin dimers; the glycosylation of Ser34 (not shown) also promotes dimer formation, but to a lesser extent. While the exact details of the dimer formation process remain to be clarified, biochemical evidence suggests that glycosylation mediates interactions between the head domain of one vimentin molecule and either the head or rod domain of a second vimentin molecule, or with assembled vimentin filaments.

Tarbet et al. showed that replacing Ser34, but not Ser39, with the amino acid alanine reduced O-GlcNAc-mediated crosslinking between vimentin molecules, and that replacing Ser49 with alanine molecules completely prevented vimentin crosslinking, whereas mutations at several other amino acids did not have a measurable effect. This represents the first evidence that O-linked glycosylation can modulate interactions between vimentin molecules, and the work was enabled by a recently established chemical labeling technique to identify O-GlcNAc targets (Yu et al., 2012).

Tarbet et al. then focused on Ser49 because it was not a known phosphorylation site: replacing this serine with either alanine (which cannot undergo phosphorylation) or with aspartic acid (which has charge properties that resemble a permanently phosphorylated serine) resulted in disrupted vimentin structures, rather than extended filaments that are usually formed. This suggests that the inability of these two mutants to form filaments is due to the loss of glycosylation or possibly, in the case of the aspartic acid mutant, due to there being a permanent phosphorylation-like state at Ser49.

Importantly, Tarbet et al. extended their findings to demonstrate that the glycosylation of Ser49 is critical for vimentin-dependent cell migration and for the replication of the bacteria *Chlamydia* inside cells after infection. When *Chlamydia* infects a normal cell it takes over the cell, forcing vimentin molecules to form cages that protect and separate the bacteria from the host cytoplasm as it replicates. However, in the presence of a glycosylation inhibitor, or when Ser49 has been made non-glycosylatable, this 'hijacking' process is reduced significantly.

A few caveats deserve mention. First, the possibility that some of the results reported are due to indirect effects of the Ser49 mutation have not been completely excluded. Second, there is some confusion about Ser49 and phosphorylation: although the PhosphoNET database reports that Ser49 is phosphorylated in vimentin, the citation for this actually corresponds to Ser51, which is known to be a phosphorylation site (Izawa and Inagaki, 2006). However, several proteomic studies in the PhosphoSitePlus database report that Ser49 on human vimentin is a phospho-site, but these reports require detailed validation.

One surprising finding was that a mutation at a single glycosylation site (Ser49) had such a disruptive effect on vimentin filament formation, whereas the simultaneous mutation of all three major glycosylation sites on another intermediate filament protein, keratin 18, did not have a comparable impact (Ku and Omary, 1995). This could be because keratin 18 forms heterodimers (with keratin 8), so the impact is buffered by the partner keratin, whereas vimentin molecules form homodimers with other vimentin molecules.

Further experiments are needed to fully understand the assembly of intermediate filaments in vivo, including the role that 'cross-talk' between glycosylation and phosphorylation plays, but the findings of Tarbet et al. have illuminated an important aspect of the process.
